# Association Between Digital Front Doors and Social Care Use for Community-Dwelling Adults in England: Cross-Sectional Study

**DOI:** 10.2196/53205

**Published:** 2025-01-02

**Authors:** Jinbao Zhang, Jonathan E Prunty, Alison C Charles, Julien Forder

**Affiliations:** 1 Personal Social Services Research Unit University of Kent Canterbury United Kingdom; 2 Leverhulme Centre for the Future of Intelligence University of Cambridge Cambridge United Kingdom; 3 Centre for Health Services Studies University of Kent Canterbury United Kingdom

**Keywords:** social care support, long term care, access, front door, easy-read, self-assessment, system navigation, digital system, digital technology, internet

## Abstract

**Background:**

Requests for public social care support can be made through an online portal. These digital “front doors” can help people navigate complex social care systems and access services. These systems can be set up in different ways, but there is little evidence about the impact of alternative arrangements. Digital front-door systems should help people better access services, particularly low-intensity services (high-intensity care is likely to require a full in-person assessment).

**Objective:**

This study aimed to investigate the association between 2 primary digital front door arrangements, easy-read information, and self-assessment tools provided on official websites, and the type of social care support that is offered: ongoing low-level support (OLLS), short-term care (STC) and long-term care (LTC).

**Methods:**

Information on front door arrangements was collected from the official websites of 152 English local authorities in 2021. We conducted a cross-sectional analysis using aggregated service use data from official government returns at the local authority level. The independent variables were derived from the policy information collected, specifically focusing on the availability of online digital easy-read information and self-assessment tools for adults and caregivers through official websites. The dependent variables were the rates of using social care support, including OLLS, STC, and LTC, across different age groups: the adult population (aged 18 and older), younger population (aged between 18 and 64 years), and older population (aged 65 and older). Multivariate regression analysis was used to examine the association between digital front door arrangements and access to social care support, controlling for population size, dependency level, and financial need factors.

**Results:**

Less than 20% (27/147) of local authorities provided an integrated digital easy-read format as part of their digital front door system with about 25% (37/147) adopting digital self-assessment within their system. We found that local authorities that offered an integrated digital easy-read information format showed higher rates of using OLLS (β coefficient=0.54; *P*=.03; but no statistically significant association with LTC and STC). The provision of an online self-assessment system was not associated with service use in the 1-year (2021) cross-sectional estimate, but when 2 years (2020 and 2021) of service-use data were analyzed, a significant positive association was found on OLLS rates (β coefficient=0.41; *P*=.21). Notably, these findings were consistent across different age groups.

**Conclusions:**

These findings are consistent with our hypothesis that digital systems with built-in easy-read and self-assessment may make access to (low-intensity) services easier for people. Adoption of these arrangements could potentially help increase the uptake of support among those who are eligible, with expected benefits for their care-related well-being. Given the limited adoption of the digital front door by local authorities in England, expanding their use could improve care-related outcomes and save social care costs.

## Introduction

### Background

The “front door” to the adult social care system refers to the channels through which people collect service information, apply for needs assessment, and access social care support. Social care systems vary in how they configure their front door and information may not always be well organized or easily accessible, and different services have specific eligibility requirements based on factors such as wealth, income, and physical care needs. Navigating the front door can be demanding and time consuming for community-dwelling people with functional impairments [1]. When requesting services, people need to proactively acquire service information and approach care professionals to complete face-to-face needs assessment [2]. Without accessible information and streamlined application procedures, individuals may be less able to access social care support, causing unmet needs to escalate [3,4]. Therefore, developing front door arrangements is crucial to ensure that people can access available and timely services, potentially preventing or delaying the escalation of care needs.

Researchers and policy makers have recently become increasingly attentive to deploying digital front door arrangements to facilitate service access. While a consensus on the definition of the digital front door is lacking, we define it as the channels through which individuals request and access services using digital platforms or technologies [5]. Digital technologies may facilitate service access in three primary ways: (1) enabling initial contact with services, (2) substituting traditional face-to-face interactions with remote services, and (3) facilitating access to professional support through innovative technologies [6]. For instance, an online information and referral tool, BenefitsCheckUp, increased the take-up rate of Medicaid among low-income older Americans [7]. In England, the Care Act 2014 has stipulated that local authorities are responsible for providing digital channels (eg, websites and social media) to help individuals make informed choices, which can also reduce public expenditure at the front door [8,9]. Despite the growing emphasis on digital front door arrangements, few studies have examined whether digital channels can effectively promote service access [6].

To fill the gap, this study explores the association between the digital front door and the use of social care support in England. Specifically, we define the digital front door as whether local authorities provide online digital easy-read information and self-assessment tools for adults and caregivers through official websites. In addition, we investigate whether access to different types of social care support is correlated with local authorities’ digital front door arrangements. This study contributes to understanding the relationship between the digital front door and service access, providing insights into how to organize these systems to facilitate access to social care support. The findings could inform policy makers about developing digital front door arrangements to promote service access, eventually enhancing people’s well-being.

### Digital Front Doors and Access to Social Care Support

People needing social care have generally relied on social networks (eg, peers and family members) and health and social care professionals as primary resources for information and assistance [1,4,10]. Since the COVID-19 pandemic, digital channels have become an increasingly viable alternative that could save costs and facilitate service access [6,8,11]. The digital front door can do this by alleviating 2 main obstacles to service use: information asymmetry and administrative burdens [2,4,10,12].

Providing online information can address information asymmetry and facilitate service access by potentially simplifying applications and allowing immediate completion. They eliminate travel costs as well as removing waiting times for printed versions of documents [13]. Previous studies have suggested that online information should be presented from trusted sources and in easy-read information formats to meet individual preferences [14,15]. Specifically, people trust information from official websites and medical professionals. Also, easy-read information is generally characterized by plain language, simple layout, large font size, and the adoption of images. Despite individual preferences for digital easy-read information from government websites, few studies have explored the relationship between such information and access to social care support.

In addition to easy-read information online information, online self-assessment tools mitigate the administrative burden associated with applying for social care support. Self-assessment aims to reduce direct professional involvement in the assessment process. Savings can arise from reduced paperwork and waiting, travel, and face-to-face time associated with professionally led needs assessment [16,17]. Self-assessment approaches could encourage access for people with low-level needs when perceived barriers to application may outweigh the potential benefits [16,18]. Although previous studies mainly highlighted the positive association between service access and self-assessment tools [16,17], little work has focused on their relationship in an online context. Potential arguments against self-assessment include a potential lack of identification of person-specific needs and idiosyncrasies [19].

### Social Care and Front Doors in England

Local authorities in England are responsible for delivering adult social care for people with care needs. In order to access care, individuals need to navigate the front door of social care systems, including gathering information on services and completing needs assessment [20]. Information is available in different formats, such as printed leaflets or brochures, conversations with professionals, and the internet (especially official websites) [21]. Though the Care Act 2014 stipulated local authorities’ responsibilities for providing multiple information formats, the proportion of older people who found it easy to obtain information had fallen from 75.2% in 2016 to 65.6% in 2021 [22]. This decline indicates the need for understanding how to provide information and alleviate information asymmetry to promote service access.

Traditionally, assessment has been performed by professionals through telephone and face-to-face communications. As of August 2022, there were half a million people waiting for a care assessment, for their care and support to begin, or for a review of their care plan [23]. By March 2023, the number of people who had been waiting for over 6 months for their care assessment rose to 82,087. Every year, local authorities can together expect around 2 million requests for care, with an average of 5420 requests for support received each day [24]. Within this context, many local authorities introduced self-assessment as a complementary tool for professionally led assessments [11,16].

People generally access publicly funded services following a hospital discharge or are referred from the community. In 2021, most (79.1%) of care requests originated from the community, while 18.7% were discharged from the hospital (with 2.2% from other routes) [24]. While the number of requests has grown during the past 7 years, the pattern of requests by route of access has largely remained unchanged.

Following the care request, people eligible for publicly funded services can receive 3 main types of support: short-term care (STC), long-term care (LTC), and other services, including end-of-life care and ongoing low-level support (OLLS) that targets community-dwelling people with minimal care needs and offers them ongoing services (eg, telecare, minicom live and community alarm).

### Study Aims

Despite the development of the digital front door in England, the relationship between these arrangements and service access remains unclear. Understanding their associations could guide policy makers and practitioners to improve front door arrangements and facilitate service use, thus improving people’s well-being. Accordingly, this study investigates the prevalence of providing easy-read information online information and self-assessment tools through official websites and the association between such digital channels and social care use.

We focus on people requesting care from the community because they are most likely to use digital front door arrangements. By contrast, for people being discharged from the hospital, there are generally different and more specific arrangements to access social care. Furthermore, people discharged from hospital are likely to have higher levels of need. Access from the community accounts for approximately 80% of all care requests, people also tend to wait longer for assessment and support (60 days) than those discharged from the hospital (38 days) [25].

## Methods

### Data

This study used upper-tier local-authority-level data from local authorities’ websites, the short and long-term (SALT) collection on care access, use, and expenditure [24], and Stat-Xplore for benefits data (Department for Work and Pensions). To identify local authorities’ digital front door arrangements, we gathered policy information from 152 local authorities’ websites between December 2022 and April 2023 and coded these documents based on established criteria. Specifically, we defined local authorities that used the “easy-read information” keyword in their official websites to introduce adult social care systems and application procedures as providing easy-read information. Those that did not have such a keyword, or did not permit immediate access through official websites, were coded as not having easy-read information. Our choice of focusing on “easy-read information” allows us to use a straightforward criterion to identify intentional effort by local authorities to provide online digital easy-read information. Likewise, local authorities that provided online self-assessment forms (excluding contact forms) were coded as having online self-assessment tools for adults and caregivers, respectively. To ensure the consistency of policy texts, 2 researchers (JZ and AC) independently coded each local authority’s digital front door arrangements. Disagreements were addressed through discussions between the 2 investigators (JZ and AC).

We also obtained information about how clients accessed care services (through the community or hospital route) and their subsequent care destination (STC, LTC, and other services) from the SALT data. This data set has been published annually since 2016 and contains information about clients’ journeys through the social care system in England, including the number of requests for social care, the access route for people requesting support, and their care sequel (what happened next, eg, community and residential care).

In addition, this study collected local area characteristics, such as population estimates and pension credit, from the Stat-Xplore website. Stat-Xplore website provides aggregated benefit data administered by the Department for Work and Pensions, including pension credit and Carer’s Allowance. When combining digital front door information with data from SALT and Stat-Xplore, we only included the latest wave (2021) to ensure that our results would be representative of current policy arrangements. Given that data was not available from 5 local authorities (Hackney, City of London, Isles of Scilly, North Northamptonshire, and West Northamptonshire), data from 147 local authorities only were used in our study. The data are available in Multimedia Appendix 1.

### Measurement

#### Dependent Variables

The outcome of interest was the rate of using social care support, including LTC, STC, and OLLS. Specifically, reablement services, an important type of STC that supports individuals to regain independence after an illness or hospital discharge, were included in our analysis. We also identified 3 types of LTC: community care, residential care, and nursing services. For each service type, we calculated rates using the number of people receiving the service as the numerator and the population aged 18 years or 65 years and older as the denominator.

#### Independent variables

We assessed local authorities’ digital front arrangements with 3 dichotomous variables, whether local authorities provided easy-read information online information regarding adult social care and whether they provided online self-assessment for both cared-for adults and for caregivers (no=0, yes=1).

#### Covariates

Population size, dependency level, and financial need factors were selected as covariates [26]. Population size was measured using the number of people (100,000) in 3 age groups: 18 years and older, between 18 years and 64 years, and 65 years or older. Proxy variables for care needs included the proportion of the older population aged 80 years and older, and the proportion of older people who received Attendance Allowance (the primary universal benefit for older people with social care needs). Financial need was assessed by the number of recipients receiving pension credit divided by the older population, and the proportion of the population receiving carer’s allowance (cash benefit for caregivers who provide care at least 35 hours per week and earn less than £139 (US $177) per week in 2023).

### Analysis

Multivariate regression analysis was performed. The main analysis used a cross-sectional ordinary least squares (OLS) estimation. The OLS approach was deemed appropriate for this analysis because our dependent variables are continuous. To correct the right-skewed distribution of the dependent variables, a natural log transformation was applied before analysis. This transformation helps ensure the normality assumption of OLS regression is satisfied, a method commonly used in previous studies [27,28]. We were limited to using SALT data from 2021 as the data for 2022 were not yet published at the time of analysis. As such, there is a small mismatch in timings between our categorization of front door arrangements and the care use data. Nonetheless, given that the rate of change for these variables is relatively slow, we argue that this is an acceptable limitation.

Listwise deletion was used to handle missing values on 2 dependent variables (ie, LTC for older people and OLLS). In both cases, less than 0.7% (1/147) of the cases were missing. Given the potential problem of multicollinearity, we conducted collinearity diagnostics. The mean variance inflation factors ranged from 1.28 to 2.49 and thus did not exceed the suggested threshold (variance inflation factors >10), indicating no evidence of multicollinearity [29].

### Ethical Considerations

This study used publicly available data aggregated at the local authority level. The data did not involve the collection of any personally identifiable information, nor did it involve direct interaction with human participants. As a result, this research did not require ethical approval from an ethics committee.

## Results

### Descriptive Statistics

Table 1 summarizes relevant characteristics of the 147 local authorities in 2021 by age group, that is, all adults aged 18 years and older, younger people aged between 18 years and 64 years, and older people aged 65 years and older. The average rate of using LTC, STC, and OLLS per 100,000 population for all adults was 4.73, 7.04, and 11.53, respectively. Approximately 20% (27/147) of local authorities provided digital, web-based easy-read information online information about adult social care systems. The proportion of local authorities that provided online digital self-assessment for adults and caregivers was 25% (37/147) and 27% (40/147), respectively. The average adult population (aged 18 years and older) across local authorities was 294,000, while the average older population (aged 65 years and older) was 63,000.

In addition, we used chi-square tests to identify whether the provision of the digital front door varied among the 9 larger administrative areas in England. The results are presented in Table S1 in Multimedia Appendix 2, which suggests significant differences across regions. Among the 9 regions, the East of England had the highest proportion of local authorities that offered easy-read information online information, and the Southeast had the highest proportion of local authorities providing online self-assessment for adults and caregivers. In contrast, local authorities in the Northeast and the Northwest did not offer easy-read information online information, and the Southwest had the lowest proportion of local authorities providing online self-assessments for adults and caregivers.

**Table 1:** Characteristics of local authorities (n = 147) in England in 2021 by age group.

Variable		Age 18 years and older, mean (SD)	Age 18 years and 64 years, mean (SD)	Age 65 years and older, mean (SD)
**Dependent variables**				
	Long-term care rate	4.73 (3.30)	3.55 (2.57)	8.92 (5.60)
	Short-term care rate	7.04 (7.36)	5.53 (6.34)	12.43 (11.39)
	Ongoing low-level support rate	11.53 (11.92)	8.90 (9.16)	20.68 (21.33)
**Independent variables**				
	Easy-read information (=1)	0.18 (0.39)	0.18 (0.39)	0.18 (0.39)
	Self-assessment for adults (=1)	0.25 (0.44)	0.25 (0.44)	0.25 (0.44)
	Self-assessment for caregivers (=1)	0.27 (0.45)	0.27 (0.45)	0.27 (0.45)
**Covariates**				
	Population size (100,000)	2.94 (2.22)	2.24 (1.63)	0.70 (0.63)
	Proportion of older people aged 80 years and older	0.27 (0.02)	—^a^	0.27 (0.02)
	Proportion of older population receiving Attendance Allowance	0.50 (0.07)	—	0.50 (0.07)
	Proportion of older population receiving pension credit	0.54 (0.24)	—	0.54 (0.24)
	Proportion of population receiving caregiver’s allowance	0.10 (0.04)	0.11 (0.04)	0.10 (0.04)

^a^Not applicable.

### Digital Front Doors and Social Care Support

Table 2 shows the association between the digital front door and the rate of using social care support. Panels 1, 2, and 3 show the estimates for LTC, STC, and OLLS, respectively. For each outcome, we investigated the association with the digital front door by three age groups: (1) all adults aged 18 years and older, (2) younger people aged between 18 years and 64 years, and (3) older people aged 65 years and above. The rate of using OLLS for all adults (β coefficient=0.54; *P*=.03), younger people (β coefficient=0.48; *P*=.04), and older people (β coefficient=0.53; *P*=.04) was positively associated with providing easy-read information online information. Providing online self-assessment tools for adults and caregivers was not significantly related to the rate of using OLLS. Providing easy-read information online information and online self-assessments for adults and caregivers were not significantly associated with LTC and STC.

Given the small sample size in our main analysis, we conducted a robustness check by also including care use data for 2020, assuming the same configuration of easy-read information and self-assessment as for 2021. The results are presented in Table S3 in Multimedia Appendix 2. We found that providing easy-read information online information was still positively associated with the rate of using OLLS, regardless of age group. However, providing online self-assessment for adults was now positively associated with the rate of using OLLS for all age groups using this larger sample.

Given that LTC and STC are general categories that encompass many types of services, we investigated the association between the digital front door and specific subdivisions of these services including restorative services, community care, residential care, and nursing care. The results of this analysis are summarized in Table S4 in Multimedia Appendix 2, together with the relationship between the digital front door and funded social care support. All associations between these types of services and providing easy-read information and online self-assessment were non-significant for both adults and caregivers.

**Table 2:** Results of multivariate regression analysis examining the association between the digital front door and social care support in England in 2021

			Age 18 years and older				Age 18 years and 64 years				Age 65 years and older		
			Values		*P* value		Values		*P* value		Values		*P* value
**Panel 1: Long-term care**													
	Easy-read information, β coefficient (SE)	–0.15 (0.15)		.32		–0.14 (0.15)		.34		–0.03 (0.10)		.78	
	Self-assessment for adults, β coefficient (SE)	0.10 (0.12)		.42		0.07 (0.13)		.56		0.08 (0.12)		.48	
	Self-assessment for caregivers, β coefficient (SE)	–0.04 (0.11)		.72		–0.01 (0.13)		.91		–0.06 (0.11)		.59	
	Covariates^a^	Yes		—^b^		Yes		—		Yes		—	
	Local authorities, n	147		—		146		—		146		—	
	*F* test (*df*)	3.66 (8)		<.001		3.63 (5)		.004		1.67 (8)		.11	
	*R^2^*	0.22		—		0.13		—		0.10		—	
**Panel 2: Short-term care**													
	Easy-read information, β coefficient (SE)	–0.32 (0.24)		.19		–0.35 (0.26)		.17		–0.30 (0.22)		.18	
	Self-assessment for adults, β coefficient (SE)	–0.21 (0.25)		.39		–0.25 (0.25)		.33		–0.19 (0.25)		.44	
	Self-assessment for caregivers, β coefficient (SE)	0.22 (0.25)		.38		0.25 (0.25)		.33		0.22 (0.26)		.39	
	Covariates	Yes		—		Yes		—		Yes		—	
	Local authorities, n	147		—		147		—		147		—	
	*F* test (*df*)	1.73 (8)		.10		2.27 (5)		.05		0.98 (8)		.11	
	*R^2^*	0.11		—		0.08		—		0.06		—	
**Panel 3: Ongoing low-level support**													
	Easy-read information, β coefficient (SE)	0.54 (0.24)		.03		0.48 (0.24)		.04		0.53 (0.25)		.04	
	Self-assessment for adults, β coefficient (SE)	0.37 (0.27)		.17		0.34 (0.28)		.23		0.35 (0.27)		.19	
	Self-assessment for caregivers, β coefficient (SE)	–0.04 (0.28)		.88		–0.03 (0.29)		.91		–0.05 (0.28)		.85	
	Covariates	Yes		—		Yes		—		Yes		—	
	Local authorities, n	146		—		146		—		146		—	
	*F* test (*df*)	6.86 (8)		<.001		8.16 (5)		<.001		4.42 (8)		<.001	
	*R^2^*	0.23		—		0.20		—		0.19		—	

^a^We presented estimators of all covariates in Table S2 in Multimedia Appendix 2.

^b^Not applicable.

## Discussion

### Principal Findings

To our knowledge, this is the first study that provides information regarding the prevalence of digital easy-read information and self-assessment tools from official websites in England and investigates the association between these tools and access to social care support. Using data at the local authority level, we found that only 20% (27/147) of local authorities provided online, digital easy-read information, and approximately 25% (37/147) used digital self-assessment approaches to promote service access. This is important as we found local authorities that provided easy-read information had a higher rate of using OLLS than those without such a front door arrangement.

### Comparison With Previous Work

Providing easy-read information has been shown to reduce learning costs and enhance comprehension [30,31]. Our study adds to this literature by demonstrating the association between easy-read information online information and service access. The availability of digital easy-read information was positively associated with the use of OLLS, but we found no significant association with rates of LTC or STC use. One possible interpretation as to why easy-read information online information only facilitated access for those eligible for low-level ongoing care is that people using web-based digital tools to access care services may have less physical and cognitive impairment, that is lower needs, than their counterparts [32,33]. Conversely, people with more severe needs may have a higher likelihood of being referred directly to a full professional assessment, bypassing the need for online access or assessment. People with lower needs are also more likely to be assessed as needing OLLS rather than long-term and STC [34].

We did not find a significant association between the availability of online self-assessment tools for adults and caregivers, and access to social care support (although alternative analysis using 2 years of data did find a positive association). In practice, the provision of an online self-assessment facility does not necessarily imply that such a system is fully used. One pilot program in England showed that about two-thirds of people requested a face-to-face professionally led assessment rather than a self-assessment, and this was especially true for those with urgent and complex needs [35]. This lower rate of online self-assessment use may explain the nonsignificant association between online self-assessment and service use. In contrast, people with less intensive needs typically prefer using a self-assessment, especially when professional assistance is readily available [16]. Therefore, policy makers could develop both professionally led assessments and online self-assessments to meet the individual preferences of service users. Also, online self-assessment could be augmented with online support from care professionals (eg, through a chat function) to further assist users.

### Limitations

This study has several limitations to consider. First, the digital front door is a broad concept with multiple policy components, which may not be fully measured. To address this challenge, we investigated commonly discussed barriers to service access and digital front door arrangements while acknowledging that some digital channels are not operationalized in this study. For example, individuals may use information and assistance from nonprofit organizations’ websites, such as Age UK and Carers UK. Third-sector websites are an essential aspect of the digital front door, and future research could examine their impact on service access. In addition, although local authorities that provide easy-read information typically incorporate the term “easy-read information,” we acknowledge that some websites offer such content without explicitly using this exact keyword.

Second, this study provides an up-to-date snapshot of the digital front door in England by offering a cross-sectional analysis of current policy practices. Information regarding the digital front door between late 2022 and early 2023 was combined with social care data from 2021 to 2022 as these were the most up-to-date statistics available at the time of analysis. As noted above, given that general changes in service use are relatively slow, we believe this limitation to be minimal. Nonetheless, we do accept the possibility that, in some cases, the 2022 and 2023 social care data may better reflect the association with current digital front door policies, leading to smaller estimated effect sizes in the current analysis.

Finally, the cross-sectional design of this study limits our ability to establish causal relationships between the digital front door and social care use. While we assumed that these digital arrangements promote access to social care, the possibility of reverse causality cannot be excluded. For example, local authorities facing high demand for OLLS may introduce online digital easy-read information to reduce service inquiries, resulting in a potentially spurious correlation between these digital systems and service use. Therefore, our findings should be interpreted as indicative associations rather than causal relationships. Future research could use longitudinal data and causal inference methods to more rigorously examine the impact of the digital front door on access to social care support.

### Strengths and Implications

Despite these limitations, this study is among the first to empirically examine the association between providing easy-read information and self-assessment tools from official websites and service access. The findings presented here could inform policy makers interested in developing digital channels for service access. They are also relevant for discussions about increasing service use, reducing unmet needs, and enhancing the well-being of service users.

Our findings have implications for policy makers and practitioners who aim to promote service use. Though digital channels have the potential to save costs, we found relatively few local authorities provided online digital easy-read information and online self-assessment tools, as noted above. Although we do not have information about the costs of implementing such a facility, our findings suggest a positive association with (low-level) service use. This association could potentially lead to lower levels of unmet need and better care-related outcomes, which may, in turn, lead to cost-savings downstream. Given that digital channels might facilitate service access, there is a case for a wider roll-out of digital easy-read information facilities, particularly where the costs of implementation are minimal.

## Appendix

Multimedia Appendix 1 Data.

Multimedia Appendix 2 Supplementary results.

## Abbreviations

LTC: long-term care
NIHR: National Institute for Health and Care Research
OLLS: ongoing low-level support
OLS: ordinary least squares
SALT: short and long term
STC: short-term care

## Data Availability

All data generated or analyzed during this study are included in this published article and its Multimedia Appendix files.
