# Supplementary material for: Association Between Digital Front Doors and Social Care Use for Community-Dwelling Adults in England: Cross-Sectional Study
Source: J Med Internet Res. 2025 Jan 2;27:e53205. doi: 10.2196/53205 (PMC11739725; doi:10.2196/53205)
Supplement: Multimedia Appendix 2 [file jmir_v27i1e53205_app2.docx]

**Table S1.** Distribution of digital front door arrangements across nine regions in 2021 (n = 147). (A chi-square test was conducted to assess whether there were significant regional differences in the adoption of these digital front door arrangements.)

| Region | Easy read (= 1) | | Self-assessment for adults (= 1) | | Self-assessment for carers (= 1) | |
| --- | --- | --- | --- | --- | --- | --- |
|  | Frequency | Proportion (%) | Frequency | Proportion (%) | Frequency | Proportion (%) |
| East Midlands | 3 | 37.50 | 1 | 12.50 | 1 | 12.50 |
| East of England | 5 | 45.45 | 3 | 27.27 | 1 | 9.09 |
| London | 8 | 25.81 | 9 | 29.03 | 11 | 35.48 |
| North East | 0 | 0.00 | 2 | 16.67 | 2 | 16.67 |
| North West | 0 | 0.00 | 3 | 13.04 | 4 | 17.39 |
| South East | 2 | 10.53 | 12 | 63.16 | 13 | 68.42 |
| South West | 5 | 35.71 | 1 | 7.14 | 1 | 7.14 |
| West Midlands | 2 | 14.29 | 2 | 14.29 | 3 | 21.43 |
| Yorkshire and The Humber | 2 | 13.33 | 4 | 26.67 | 4 | 26.67 |
| England | 27 | 18.37 | 37 | 25.17 | 40 | 27.21 |
| Chi-square | *χ^2^*_(8)_ =20.35, *P* = .009 | | *χ^2^*_(8)_ =21.08, *P* = .007 | | *χ^2^*_(8)_ =24.94, *P* = .002 | |

**Table S2.** Results of multivariate regression analysis examining the association between the digital front door and social care support in England in 2021. (This table reports estimates for all covariates.)

|  | **a) 18+** | | **b) 18-64** | | **c) 65+** | |
| --- | --- | --- | --- | --- | --- | --- |
|  | **b/SE** | ***P* value** | **b/SE** | ***P* value** | **b/SE** | ***P* value** |
| **Panel 1: Long-term care** |  |  |  |  |  |  |
| Easy read | -0.15 | .317 | -0.14 | .339 | -0.03 | .777 |
|  | (0.15) |  | (0.15) |  | (0.10) |  |
| Self-assessment for adults | 0.10 | .415 | 0.07 | .563 | 0.08 | .475 |
|  | (0.12) |  | (0.13) |  | (0.12) |  |
| Self-assessment for carers | -0.04 | .724 | -0.01 | .913 | -0.06 | .585 |
|  | (0.11) |  | (0.13) |  | (0.11) |  |
| Population size (100,000) | 0.01 | .753 | 0.03 | .303 | 0.01 | .870 |
|  | (0.02) |  | (0.03) |  | (0.07) |  |
| Proportion of older people aged 80 and above | 0.70 | .828 |  |  | 0.17 | .956 |
|  | (3.23) |  |  |  | (3.06) |  |
| Proportion of older population receiving Attendance Allowance | 3.95 | .611 |  |  | 0.56 | .916 |
|  | (7.76) |  |  |  | (5.33) |  |
| Proportion of older population receiving pension credit | -4.20 | .044 |  |  | 0.56 | .677 |
|  | (2.07) |  |  |  | (1.33) |  |
| Proportion of population receiving Carer’s Allowance | 26.35 | .011 | 24.26 | <.001 | 15.54 | .076 |
|  | (10.17) |  | (6.11) |  | (8.70) |  |
| n | 147 |  | 146 |  | 146 |  |
| Degree of freedom (*df*) | 8 |  | 5 |  | 8 |  |
| *F* statistics | 3.66 | <.001 | 3.63 | .004 | 1.67 | .110 |
| R^2^ | 0.22 |  | 0.13 |  | 0.10 |  |
| **Panel 2: Short-term care** |  |  |  |  |  |  |
| Easy read | -0.32 | .192 | -0.35 | .170 | -0.30 | .181 |
|  | (0.24) |  | (0.26) |  | (0.22) |  |
| Self-assessment for adults | -0.21 | .387 | -0.25 | .326 | -0.19 | .435 |
|  | (0.25) |  | (0.25) |  | (0.25) |  |
| Self-assessment for carers | 0.22 | .375 | 0.25 | .331 | 0.22 | .394 |
|  | (0.25) |  | (0.25) |  | (0.26) |  |
| Population size (100,000) | 0.03 | .398 | 0.06 | .180 | 0.08 | .472 |
|  | (0.03) |  | (0.04) |  | (0.12) |  |
| Proportion of older people aged 80 and above | -3.41 | .569 |  |  | -7.15 | .245 |
|  | (5.98) |  |  |  | (6.12) |  |
| Proportion of older population receiving Attendance Allowance | 11.59 | .238 |  |  | 19.88 | .064 |
|  | (9.79) |  |  |  | (10.66) |  |
| Proportion of older population receiving pension credit | -5.47 | .022 |  |  | -2.48 | .301 |
|  | (2.35) |  |  |  | (2.39) |  |
| Proportion of population receiving Carer’s Allowance | 11.48 | .439 | 23.17 | .013 | -21.04 | .234 |
|  | (14.80) |  | (9.21) |  | (17.59) |  |
| n | 147 |  | 147 |  | 147 |  |
| Degree of freedom (*df*) | 8 |  | 5 |  | 8 |  |
| *F* statistics | 1.73 | .098 | 2.27 | .051 | 0.98 | .111 |
| R^2^ | 0.11 |  | 0.08 |  | 0.06 |  |
| **Panel 3: Ongoing low-level support** |  |  |  |  |  |  |
| Easy read | 0.54 | .029 | 0.48 | .041 | 0.53 | .036 |
|  | (0.24) |  | (0.24) |  | (0.25) |  |
| Self-assessment for adults | 0.37 | .174 | 0.34 | .228 | 0.35 | .192 |
|  | (0.27) |  | (0.28) |  | (0.27) |  |
| Self-assessment for carers | -0.04 | .878 | -0.03 | .909 | -0.05 | .854 |
|  | (0.28) |  | (0.29) |  | (0.28) |  |
| Population size (100,000) | -0.11 | .040 | -0.12 | .086 | -0.59 | .018 |
|  | (0.06) |  | (0.07) |  | (0.25) |  |
| Proportion of older people aged 80 and above | -7.62 | .242 |  |  | -9.92 | .161 |
|  | (6.48) |  |  |  | (7.03) |  |
| Proportion of older population receiving Attendance Allowance | 4.92 | .633 |  |  | 12.31 | .263 |
|  | (10.27) |  |  |  | (10.95) |  |
| Proportion of older population receiving pension credit | -5.41 | .027 |  |  | -5.23 | .043 |
|  | (2.42) |  |  |  | (2.56) |  |
| Proportion of population receiving Carer’s Allowance | 56.60 | <.001 | 57.36 | <.001 | 31.18 | .048 |
|  | (15.68) |  | (9.61) |  | (15.61) |  |
| n | 146 |  | 146 |  | 146 |  |
| Degree of freedom (*df*) | 8 |  | 5 |  | 8 |  |
| *F* statistics | 6.86 | <.001 | 8.16 | <.001 | 4.42 | <.001 |
| R^2^ | 0.23 |  | 0.20 |  | 0.19 |  |

**Table S3.** Robustness check: Results of multivariate regression analysis examining the association between the digital front door and social care support in 2020 and 2021.

| **Panel 1: Long-term care** | **a) 18+** | | **b) 18-64** | | **c) 65+** | |
| --- | --- | --- | --- | --- | --- | --- |
|  | **b/SE** | ***P* value** | **b/SE** | ***P* value** | **b/SE** | ***P* value** |
| Easy read | -0.15 | .143 | -0.12 | .223 | -0.10 | .281 |
|  | (0.10) |  | (0.10) |  | (0.09) |  |
| Self-assessment for adults | 0.04 | .633 | 0.02 | .797 | 0.05 | .584 |
|  | (0.09) |  | (0.09) |  | (0.09) |  |
| Self-assessment for carers | -0.02 | .814 | -0.00 | .995 | -0.01 | .884 |
|  | (0.08) |  | (0.09) |  | (0.09) |  |
| Covariates | Yes |  | Yes |  | Yes |  |
| n | 294 |  | 294 |  | 293 |  |
| Degree of freedom (*df*) | 9 |  | 6 |  | 9 |  |
| *F* statistics | 5.24 | <.001 | 5.52 | <.001 | 2.62 | .006 |
| R^2^ | 0.20 |  | 0.12 |  | 0.10 |  |
| **Panel 2: Short-term care** |  |  |  |  |  |  |
| Easy read | -0.26 | .141 | -0.28 | .125 | -0.14 | .326 |
|  | (0.18) |  | (0.18) |  | (0.15) |  |
| Self-assessment for adults | -0.24 | .173 | -0.26 | .140 | -0.18 | .291 |
|  | (0.18) |  | (0.18) |  | (0.17) |  |
| Self-assessment for carers | 0.23 | .180 | 0.23 | .171 | 0.29 | .100 |
|  | (0.17) |  | (0.17) |  | (0.17) |  |
| Covariates | Yes |  | Yes |  | Yes |  |
| n | 294 |  | 294 |  | 293 |  |
| Degree of freedom (*df*) | 9 |  | 6 |  | 9 |  |
| *F* statistics | 3.25 | <.001 | 4.29 | <.001 | 1.77 | .074 |
| R^2^ | 0.12 |  | 0.08 |  | 0.05 |  |
| **Panel 3: Ongoing low-level support** |  |  |  |  |  |  |
| Easy read | 0.51 | .002 | 0.48 | .003 | 0.49 | .003 |
|  | (0.16) |  | (0.16) |  | (0.16) |  |
| Self-assessment for adults | 0.41 | .021 | 0.40 | .034 | 0.39 | .028 |
|  | (0.18) |  | (0.19) |  | (0.18) |  |
| Self-assessment for carers | -0.14 | .442 | -0.13 | .497 | -0.15 | .413 |
|  | (0.19) |  | (0.20) |  | (0.18) |  |
| Covariates | Yes |  | Yes |  | Yes |  |
| n | 292 |  | 292 |  | 292 |  |
| Degree of freedom (*df*) | 9 |  | 6 |  | 9 |  |
| *F* statistics | 10.59 | <.001 | 12.28 | <.001 | 7.17 | <.001 |
| R^2^ | 0.20 |  | 0.18 |  | 0.17 |  |

**Table S4.** Results of multivariate regression analysis examining the association between the digital front door and reablement, community, residential, nursing and social care support in England in 2021.

|  | **a) 18+** | | **b) 18-64** | | **c) 65+** | |
| --- | --- | --- | --- | --- | --- | --- |
|  | **b/SE** | ***P* value** | **b/SE** | ***P* value** | **b/SE** | ***P* value** |
| **Panel 1: Reablement** |  |  |  |  |  |  |
| Easy read | -0.13 | .634 | -0.14 | .608 | -0.11 | .667 |
|  | (0.27) |  | (0.27) |  | (0.26) |  |
| Self-assessment for adults | -0.05 | .858 | -0.07 | .778 | 0.01 | .953 |
|  | (0.26) |  | (0.26) |  | (0.25) |  |
| Self-assessment for carers | -0.04 | .889 | -0.02 | .944 | 0.00 | .997 |
|  | (0.25) |  | (0.26) |  | (0.25) |  |
| Covariates | Yes |  | Yes |  | Yes |  |
| n | 144 |  | 144 |  | 143 |  |
| Degree of freedom (*df*) | 8 |  | 5 |  | 8 |  |
| *F* statistics | 1.02 | .426 | 1.68 | .144 | 0.67 | .718 |
| R^2^ | 0.05 |  | 0.05 |  | 0.05 |  |
| **Panel 2: Community care** |  |  |  |  |  |  |
| Easy read | -0.17 | .288 | -0.16 | .284 | -0.04 | .694 |
|  | (0.16) |  | (0.15) |  | (0.11) |  |
| Self-assessment for adults | 0.12 | .349 | 0.10 | .458 | 0.11 | .400 |
|  | (0.13) |  | (0.13) |  | (0.13) |  |
| Self-assessment for carers | -0.06 | .630 | -0.03 | .790 | -0.09 | .487 |
|  | (0.12) |  | (0.13) |  | (0.12) |  |
| Covariates | Yes |  | Yes |  | Yes |  |
| n | 147 |  | 147 |  | 146 |  |
| Degree of freedom (*df*) | 8 |  | 5 |  | 8 |  |
| *F* statistics | 2.72 | .008 | 3.51 | .005 | 2.01 | .049 |
| R^2^ | 0.17 |  | 0.12 |  | 0.10 |  |
| **Panel 3: Residential care** |  |  |  |  |  |  |
| Easy read | 0.00 | .992 | 0.02 | .917 | -0.01 | .897 |
|  | (0.11) |  | (0.17) |  | (0.11) |  |
| Self-assessment for adults | -0.05 | .775 | -0.10 | .667 | -0.04 | .800 |
|  | (0.18) |  | (0.23) |  | (0.17) |  |
| Self-assessment for carers | 0.10 | .551 | 0.17 | .447 | 0.09 | .608 |
|  | (0.17) |  | (0.23) |  | (0.17) |  |
| Covariates | Yes |  | Yes |  | Yes |  |
| n | 139 |  | 139 |  | 139 |  |
| Degree of freedom (*df*) | 8 |  | 5 |  | 8 |  |
| *F* statistics | 18.35 | <.001 | 5.24 | <.001 | 7.54 | <.001 |
| R^2^ | 0.42 |  | 0.16 |  | 0.21 |  |
| **Panel 4: Nursing care** |  |  |  |  |  |  |
| Easy read | -0.23 | .208 | -0.20 | .322 | -0.19 | .271 |
|  | (0.18) |  | (0.20) |  | (0.17) |  |
| Self-assessment for adults | -0.05 | .768 | -0.22 | .297 | -0.01 | .962 |
|  | (0.18) |  | (0.21) |  | (0.19) |  |
| Self-assessment for carers | 0.00 | .985 | 0.15 | .498 | 0.01 | .963 |
|  | (0.19) |  | (0.22) |  | (0.20) |  |
| Covariates | Yes |  | Yes |  | Yes |  |
| n | 129 |  | 129 |  | 129 |  |
| Degree of freedom (*df*) | 8 |  | 5 |  | 8 |  |
| *F* statistics | 4.77 | <.001 | 1.84 | .110 | 1.30 | .250 |
| R^2^ | 0.18 |  | 0.05 |  | 0.05 |  |
| **Panel 5: Social care** |  |  |  |  |  |  |
| Easy read | -0.07 | .421 | -0.11 | .317 | -0.04 | .619 |
|  | (0.09) |  | (0.11) |  | (0.08) |  |
| Self-assessment for adults | -0.03 | .742 | -0.04 | .716 | -0.06 | .564 |
|  | (0.10) |  | (0.12) |  | (0.10) |  |
| Self-assessment for carers | 0.05 | .649 | 0.06 | .590 | 0.07 | .469 |
|  | (0.10) |  | (0.12) |  | (0.10) |  |
| Covariates | Yes |  | Yes |  | Yes |  |
| n | 147 |  | 147 |  | 147 |  |
| Degree of freedom (*df*) | 8 |  | 5 |  | 8 |  |
| *F* statistics | 11.73 | <.001 | 8.29 | <.001 | 2.02 | .049 |
| R^2^ | 0.36 |  | 0.21 |  | 0.11 |  |
